# A Bibliographic Review of Airborne Fungal Allergens from Dominant and Undercharacterized Genera

**DOI:** 10.3390/medicina62061186

**Published:** 2026-06-18

**Authors:** Noemi-Teofana Musta, Nicoleta Ianovici

**Affiliations:** Biology Department, Environmental Biology and Biomonitoring Research Center, Faculty of Chemistry, Biology, Geography, West University of Timisoara, 300223 Timișoara, Romania; noemi.musta@e-uvt.ro

**Keywords:** fungal allergy, fungal airborne allergens, IgE-mediated hypersensitivity, aerobiology

## Abstract

Atmospheric fungi represent an important group of allergens with a major impact on public health, especially among sensitized or immunocompromised individuals. This article reviews the ubiquitous fungal taxa in the atmosphere—*Alternaria*, *Cladosporium*, *Aspergillus*, *Penicillium*—as well as the undercharacterized taxa—*Epicoccum*, *Pithomyces*, *Torula* and *Rhodotorula*—with an emphasis on the antigenic composition and protein structures involved in type I hypersensitivity reactions. Recent data from the scientific literature (2000–2025) is presented, along with the frequency of spores in the atmosphere and the global distribution of research, highlighted by the analysis of Google Scholar and PubMed results. While *Alternaria*, *Aspergillus*, *Penicillium*, and *Cladosporium* are recognized as major contributors to allergic sensitization worldwide, *Epicoccum*, *Torula*, *Rhodotorula*, and *Pithomyces* remain relatively undercharacterized, but associated with allergic reactions and cross-reactivity.

## 1. Ecological and Climatic Context

This review aims to analyze the literature published between 2000 and 2025, regarding both dominant and emerging fungal genera in the atmosphere—*Alternaria*, *Penicillium*, *Aspergillus*, *Cladosporium*, *Epicoccum*, *Torula*, *Rhodotorula*, *Pithomyces*. While *Alternaria*, *Aspergillus*, *Penicillium*, and *Cladosporium* are recognized as major contributors to allergic sensitization worldwide, *Epicoccum*, *Torula*, *Rhodotorula*, and *Pithomyces* remain relatively undercharacterized, but are associated with allergic reactions and cross-reactivity. To ensure a rigorous and up-to-date overview, a systematic search of the PubMed and Google Scholar databases was performed, using combinations of keywords related to the selected fungal genera.

In recent decades, the problems of climate and chemical changes followed by their effects on human health have been a subject of interest for both the general public and the scientific community. Bioaerosols can represent up to 10% of the total mass of suspended particles in the atmosphere [1]. Among these particles are fungal spores. The concentration of spores in the atmosphere depends on environmental factors (temperature, wind, humidity, precipitation), and the amplitude of the action on health depends on the air pollutants [2,3]. Epidemiological studies have shown that the Westernized lifestyle, urbanization and high levels of emissions resulting from the combustion of fossil fuels are correlated with an increased frequency of respiratory allergies, predominantly in the urban population compared to the rural one, likely due to the combined effects of air pollutants and airborne allergens in enhancing airway inflammation and allergic sensitization [4].

From a climatic point of view, the 21st century represents a peak of global warming. In addition to the increase in average temperature, extreme weather events such as cyclones, droughts and hurricanes, and the frequency of heat waves are on the rise. According to the World Health Organization, the predominantly negative effects of these changes are reflected in drinking water, stable food sources and air quality in both outdoor and indoor environments [5,6]. By favoring periods of drought and atmospheric dryness, global warming has led to significant changes in the spectrum of fungal taxa present in the atmosphere and in the biochemical transformations they undergo [7]. Smoke from wildfires can damage epithelial integrity, with subsequent effects on T helper lymphocytes [8]. Multiple cases of asthma attacks during thunderstorms have been documented due to the disintegration of pollen grains, resulting in the release of allergenic particles [9]. The study by Pulimood et al., 2007 [10] in the UK demonstrated that asthma patients sensitized to the release of aeroallergens during these storms were prone to develop respiratory allergies to *Cladosporium* and *Alternaria* spores.

Air pollutants can modify the allergenic potential of fungal spores by chemical modifications, increasing their allergenic potential and facilitating their entry into the respiratory tract. Direct action on the body, causing inflammation, oxidative stress and damage to the respiratory epithelium, can culminate in an allergic response [11]. Among the substances that have a significant impact on air quality are carbon dioxide, particulate matter, nitrogen dioxide, ozone and sulfur dioxide [12]. The number of fungal spores increases with the concentration of carbon dioxide in the atmosphere, which leads to an amplification of the presence and severity of respiratory allergies [7]. Through the action of nitrogen dioxide, ozone, sulfur dioxide and particulate matter, the epithelial barrier becomes more permeable, immune responses are modified and dysbiosis occurs [12,13,14,15]. The association between pollution and heat stress increases morbidity and mortality by inducing inflammation and lowering the threshold of allergic airway reactivity [16].

Environmental and climatic changes may also influence the distribution and prevalence of atmospheric fungal taxa, potentially modifying the patterns of both exposure and sensitization. While dominant genera are habituated to urban and polluted environments, changing ecological conditions may additionally favor the spread of lesser explored fungi. Variations in temperature, humidity and atmospheric carbon dioxide concentrations can influence sporulation and seasonal distribution, leading to changes in airborne spore load and exposure duration. In this context, prolonged exposure to mixed airborne fungal allergens may contribute not only to the amplification of inflammation, but also to the emergence of sensitization patterns that are currently insufficiently understood.

## 2. Relevant Immunological Aspects

Allergic reactions occur when a genetically susceptible individual interacts with a trigger factor, which has a broad spectrum of action, affecting people of different ages and multiple organs [17]. Trigger factors are antigens of different types and sizes, depending on how they are recognized by the immune system: low molecular weight proteins can associate with other serum proteins to form epitopes, and high molecular weight proteins can be recognized and processed directly by antibodies and T lymphocytes [18].

Fungi are eukaryotic, heterotrophic organisms, ubiquitous in nature, widespread in different habitats and with a dual role as symbionts and saprophytes [19,20]. The body of fungi is called mycelium and is made up of hyphae [21]. Reproduction occurs through spores, sexual or asexual, which can easily disperse in the atmosphere [22,23]. They are among the first structures identified as having hypersensitivity effects on the body. The incidence of allergies caused by fungi has increased in the last two decades [24,25].

Fungi produce proteins that, once they enter the body, cause the formation of specific IgE antibodies and, implicitly, sensitization. Subsequent exposure to the same antigens will lead to the appearance of an allergic reaction, generally manifested by allergic rhinitis and asthma [26]. Studies indicate a clear link between exposure to airborne fungal spores and hypersensitivity reactions of the body. The severity of the reactions produced in the body upon the penetration of proteins with an antigenic effect is increased: unlike other allergens, fungal spores can lead to the appearance of severe complications that endanger the lives of immunocompromised patients [27]. Next, fungal genera whose role in hypersensitivity has been proven will be presented.

### 2.1. Alternaria

*Alternaria* is a genus belonging to the class *Dothideomycetes*. The first identified species is *Alternaria alternata*, whose original name was *A. tenuis*, followed by *Torula alternata* [28]. It contains saprophytic and pathogenic species that live mainly in the atmosphere, but also on soil, in indoor environments or on plants. Most *Alternaria* species are involved in the decomposition of organic matter and can act as pathogens for humans, animals and plants. As a phytopathogen, it can lead to crop damage both before and after harvest. In humans and animals, the species *A. alternata* leads to toxicosis, due to the toxic metabolites it produces (perylene, dibenzopyrone) and to allergic reactions, mainly through the Alt a 1 protein [29,30].

The incidence of sensitization to *A. alternata* is high, making it difficult to estimate an exact percentage. Studies on the effects of sensitization on the body are numerous and diverse, indicating the importance of studying the genus. The GA(2)LEN study identified a prevalence of symptoms of 69% in European patients, and it is well known that allergic reactions caused by *A. alternata* frequently require medical care and can lead to asthma [31]. In the United States, a direct link between the presence of spores in homes and the onset or exacerbation of asthma symptoms has been demonstrated [32]. The study by Downs et al., 2001 [33] demonstrated that, in children sensitized with *Alternaria*, wheezing and airway reactivity were higher than in non-sensitized children. The action of the fungus on the innate immune system has as possible consequences increased cathelicidin levels and mucus hypersecretion [34]. Repeated administration of *A. alternata* to murine models infected with influenza virus has been shown to cause, in addition to nasal mucosal inflammation, unexpected increases in morbidity and mortality [35]. Another study also in murine models demonstrates the importance of the major allergen Alt a 1, which can induce airway inflammation and allergic asthma [36].

In order to decipher the clinical profile, it was necessary to identify the allergenic proteins from *A. alternata*, describe them and identify homologues in other species to explain the phenomenon of cross-reactivity. In total, 17 proteins with a potential allergenic role were characterized, of which 12 are considered to be of increased importance [29,37]. The main possible allergens, presented in Table 1, were retrieved from the database www.allergen.org [38]. The list of allergens includes proteins ubiquitous in the environment, conserved in the evolutionary process, with homologues within species of the genera *Aspergillus*, *Penicillium*, and *Cladosporium* [39]. The presence of homologs indicates a high degree of cross-reactivity [40], the consequence of which is clinically observable by the occurrence of allergic reactions to multiple fungal proteins in patients with sensitivity to *Alternaria* [41]. Also, all the structures associated with *A. alternata* presented in the table are airborne. Complementary to www.allergen.org is the database www.allergome.com [42]. The difference between the two is the rigor with which the structures are registered, so that the www.allergen.org database indicates only the structures whose allergenicity has been demonstrated, while www.allergome.com includes all the structures that could represent an allergen [43]. This database also includes Alt a 2, an allergen excluded due to contradictory results obtained in clinical studies, and Alt a 9, whose action is still incompletely elucidated [43,44,45].

### 2.2. Cladosporium

The genus *Cladosporium* comprises over 750 species of fungi distributed in various indoor and outdoor habitats, disseminating spores in aquatic, terrestrial and atmospheric environments. In addition to their allergenic potential, the fungi can act as phytopathogens and, in immunocompromised individuals, can be agents triggering opportunistic infections [55,56]. Spores from fungi of this genus tend to dominate bioaerosols in the atmospheric environment, with concentrations ranging from 1 to 10,000 spores/cubic meter of air. Once inhaled, they lead to irritation of the respiratory tract and the onset of respiratory allergies [57].

The two most important species involved in triggering hypersensitivity reactions are *Cladosporium herbarum* and *Cladosporium cladosporioides*. *C. herbarum* is a common contaminant in clinical laboratories and has been associated with various diseases, including pulmonary mycotoxicosis. *C. cladosporioides*, a common saprophytic species, can cause skin and lung infections. However, this species also has beneficial properties, acting as an antibiotic agent against microorganisms such as *Escherichia coli*, *Bacillus subtilis* and *Candida albicans*, and having possible insecticidal effects [58]. Table 2 presents the main clinically relevant allergens retrieved from the www.allergen.org database, grouped according to origin. From a biochemical point of view, the structures involved in the allergenicity of fungi of the genus *Cladosporium* are enzymes (vacuolar serine protease, transaldolase, enolase, mannitol dehydrogenase, aldehyde dehydrogenase) or proteins (glycoprotein, ribosomal proteins P1 and P2, YCP4 protein).

### 2.3. Aspergillus

The genus *Aspergillus* comprises over 200 species—339 known, of which 20 are pathogenic to humans. They are saprophytes and have their habitat in the soil [65,66]. Among the filamentous fungi, species belonging to the genus *Aspergillus* have been frequently associated with the induction of asthma and other allergic respiratory problems. More than 80% of these diseases are caused by *Aspergillus fumigatus* [27]. The exact mechanism behind allergic airway diseases induced by *A. fumigatus* remains uncertain, although there are theories according to which the mycelium and conidia of *Aspergillus* may persist in the airways long enough to release antigens that affect ciliary function and/or lead to damage to the lung structure [67]. The first cells to come into contact with inhaled conidia are the bronchial epithelial cells, which secrete antimicrobial compounds to protect the airways [68]. At this level, *Aspergillus* secretes sialic acid residues, which are thought to be able to shape the airway epithelium, as studies show that pathogenic *Aspergillus* species have a higher concentration of sialic acid than non-pathogenic species [69]. The airway epithelium is, in this case, a factor in the pathogenesis of asthma and allergic diseases [67]. In addition to allergic bronchopulmonary aspergillosis (ABPA), *Aspergillus* is known to produce toxins such as aflatoxin and gliotoxin. Gliotoxin promotes fungal entry into lung epithelial cells and suppresses host immune responses, and aflatoxin is ingested through contaminated food [70].

*Aspergillus* species with allergenic potential are *Aspergillus niger*, *Aspergillus flavus*, *Aspergillus versicolor*, *Aspergillus oryzae*. Table 3 presents a selection of the main allergenic substances of the listed species, recognized as having clinical relevance, retrieved from the database www.allergen.org. The proteins, enzymes and the FG-GAP domain are ordered according to their origin. The descriptions focus on allergenicity in the context of asthma or ABPA, an inflammatory disease caused by immunological reactions that occur when immunocompromised patients’ airways are colonized with *A. fumigatus* [71]. There are also notes regarding the potential for cross-reactivity.

### 2.4. Penicillium

The genus *Penicillium* comprises over 100 species, occurring in a wide range of habitats, including air, extreme environments (in terms of salinity, temperature, pH variation and water scarcity), soil and various food products. *Penicillium* contaminates food and colonizes humid indoor environments. Due to their diversity and ability to survive in extreme environments, fungi have considerable potential for various applications in various fields. From a medical point of view, the genus is of particular importance due to penicillin, a molecule used as an antibiotic. Other species are used in the food industry, especially in the dairy industry [92,93].

*Penicillium* spores are among the most widespread aeroallergens, and their presence in damp buildings is a risk factor for asthma [56]. Studies conducted in the Taipei and Topeka areas have shown that the main species identified in homes, and therefore the species for which the study of the main allergens is necessary, are: *P. citrinum*, *P. oxalicum*, *P. chrysogenum* (*P. notatum*), *P. spinulosum*, *P. brevicompactum* [94]. A selection of the main allergenic substances of the genus *Penicillium* is presented in Table 4, retrieved from the database www.allergen.org. The structures have been grouped according to origin, and the description takes into account IgE reactivity or cross-reactivity.

### 2.5. Epicoccum

*Epicoccum nigrum* (syn. *E. purpurascens*) is a common phytopathogen; in the outdoor environment it can be identified as a saprophyte of soil and plant debris, as well as an epiphyte on leaves. In indoor environments, it is found in dust and damp materials. The genus includes asexual fungi of the order *Pleosporales*. The spores are easily identified in air samples and, like *Alternaria* spores, show an increased concentration between late summer and early autumn. *Epicoccum* can cause allergic-mediated respiratory conditions, including allergic fungal sinusitis and hypersensitivity pneumonitis [56,105,106].

In the database www.allergen.org, only one protein with an allergen role is identified, namely Epi p 1. Serine protease with a molecular mass of 30 kDa, was purified from the species *E. nigrum*. It is recognized by all patients who show hypersensitivity to *Epicoccum* and cross-reacts with other fungal proteins, including those from the genus *Alternaria* [107,108].

### 2.6. Pithomyces/Pseudopithomyces

The genus *Pithomyces* includes *P. chartarum*, a saprophytic fungus found in soil, atmosphere, and vegetation, with both toxic and non-toxic strains. In humans, the fungus can cause onychomycosis and, through inhalation of spores, asthma [109]. *Pithomyces* spores can produce mycotoxins with toxic potential and possible allergic impact [110,111]. No allergen structures are registered in the www.allergen.org database, but the www.allergome.com database contains the allergen Pit m—from *Pseudopithomyces maydicus*—whose presence can be identified by the HIA (Halogen Immunoassay) technique [112]. There is also cross-reactivity between Pit m and Alt a 1 [60].

### 2.7. Torula and Rhodotorula

Torula is a genus of asexual fungi in the family *Torulaceae*, *Ascomycota*, generally saprophytic and ubiquitous in nature. It is found in moist habitats. The spores are light green or brown, have thick walls with a complex structure and a smooth surface [113,114]. *Torula* is frequently associated with type I allergies, causing asthma and allergic rhinitis [115]. There are no isolated allergenic structures belonging to this genus.

The genus *Rhodotorula*, belonging to the phylum *Basidiomycota*, comprises yeasts present in air, aquatic environments, soil, milk and fruit juices. The fact that species of the genus belonging to the phylum *Basidiomycota* can colonize humans, plants and other mammals was known, but the first infections in humans were documented after 1985 [116,117].

In addition to central nervous system infections, ocular infections, endocarditis, and peritonitis, *R. mucilaginosa* can cause hypersensitivity reactions [56,117]. The two allergen structures described in the www.allergen.org database are Rho m 1 and Rho m 2.

Rho m 1 is an enolase with a molecular weight of 47 kDa. Chang et al. study found that enolase-bound immunoglobulin E was identified in the serum of 21% of patients allergic to *R. mucilaginosa* [118]. Rho m 2 is a vacuolar serine protease with a molecular weight of 31 kDa. The same study found that immunoglobulin E against Rho m 2 was identified in the serum of 57% of patients with bronchial asthma allergic to *R. mucilaginosa* [119].

As it can be seen in this chapter, allergenic proteins from dominant atmospheric fungi are well characterized (Alt a 1 from *A. alternata*, as well as allergens from *Aspergillus*, *Penicillium*, and *Cladosporium* species). Numerous allergenic proteins have been described, including highly prevalent cross-reactive proteins such as enolases, ribosomal proteins, and serine proteases. The identification of the homologous and cross-reactive proteins shared among the characterized taxa suggests that allergic responses associated to common airborne fungi may overlap. In contrast to the dominant fungi, however, data regarding the molecular structures, sensitization pathways, and immunological action of the emerging atmospheric genera remain highly limited. Although the allergenic structures of genera such as *Torula*, *Rhodotorula*, *Epicoccum* and *Pithomyces* are less characterized, their presence in atmosphere and their capacity to induce hypersensitivity reactions suggest a possible underrecognized clinical relevance. Therefore, further studies are needed in order to better understand whether these genera represent independent sensitizing agents or their contribution is linked to the cross-reactive structures they share with the dominant taxa.

## 3. Modern Diagnostics and Methodologies

Since the 19th century, the importance of monitoring the composition of indoor and outdoor air has been recognized for the diagnosis, screening and treatment of allergic patients. The first method used to quantify fungal spores was cultivation and microscopic analysis. Although still widely used, there are a number of shortcomings: the method is selective, since only taxa that can grow on standard culture media can be detected. Also, only viable spores can be cultured. Another drawback is the difficulty of distinguishing taxa with common characteristics by simple optical analysis [120]. To overcome these difficulties, new techniques have been studied and described in the literature.

Airborne particles can be quantified by passive or active methods [121]. The passive method, based on gravity (sedimentation), consists of collecting biological material on a Petri dish or on a glass slide exposed to a determined environment for a fixed period of time. Although it is simple to apply, the technique has major limitations, since it does not allow the concentration to be reported to a standardized air volume and is influenced by the shape and size of the fungal spores, small aeroallergens being difficult to capture [122]. In contrast, active volumetric methods allow the precise quantification of small particles reported to the air volume by means of filtration, impaction and impingement devices [123,124,125]. Separation from the air in the volumetric method is based on the inertia of the particles. Depending on the particle storage environment, the devices can be of the impactor (solid medium) or impinger (liquid medium) type [121]. They collect microorganisms either selective, on culture media that require incubation, or nonselective, on adhesive surfaces that allow direct microscopic analysis of viable and nonviable particles, providing a rapid insight into air quality. Filtering devices retain particles on specific membranes for subsequent analysis, the efficiency of which is directly dependent on the type of filter and pore size [122,126]. While outdoor environments are monitored standardized by Hirst-type traps to capture seasonal and circadian dynamics, in indoor environments cascade impactors (e.g., Andersen impactors) or filtration systems are preferred, capable of identifying local sources of contamination and assessing air quality in enclosed spaces [122,127].

The enzyme-linked immunosorbent assay (ELISA) technique uses the properties of enzymes to detect and quantify antigen-antibody reactions through color changes [128]. Currently, this technique is successfully used to detect and quantify major allergenic proteins such as Alt a 1 and Asp f 1 [129]. The study by Sander et al., 2012 [130], demonstrated the potential of ELISA to measure airborne fungal spores and differentiate taxa. However, there are limitations related to the lack of standardized extracts [131].

The FTIR (Fourier transform infrared) technique is a noninvasive method for analyzing biological samples that quantifies the natural vibrations of molecular bonds, measurable by IR spectroscopy. Thus, information on the molecular composition is obtained without damaging the analyzed specimen [132]. The study conducted by Zimmermann et al., 2015 [133], aimed to study fungal spores and pollen using FTIR. Clear differentiations were obtained between species and between pollen and spores. Preserved spores were analyzed, the results indicating the preservation of the chemical composition.

The fHIA (fluorescent halogen immunoassay) technique is based on the principle of immunofluorescence. The study by Green et al., 2009 [134], highlights the usefulness of the technique because multiple aeroallergens have been characterized and a broader spectrum of fungi that can induce allergic reactions has been identified. The combination of serological and environmental analyses proposed by the authors provides a broad picture of the allergens to which the patient is exposed and can significantly improve the clinical course.

Another emerging and modern diagnostic method is the use of biosensors, analytical instruments that detect and quantify specific substances by coupling a biomolecule to a transducer. Their advantages include real-time monitoring, increased specificity, and sensitivity [135]. Future directions for this technology include the integration of artificial intelligence for data interpretation [136]. A synthesis of the methods is described in Table 5, along with the advantages and limitations.

Allergen immunotherapy (AIT) is a method of treating hypersensitivity that has been used since the early 1900s [137]. The term “allergen extract” refers to glycoproteins or proteins that have not yet been processed in therapeutic treatment, as it is prepared specifically for each patient to reduce the risk of contamination [138]. The issue of standardizing extracts has been long debated, as natural products are more difficult to compare than synthetic ones [139].

The first step in standardization is to ensure quality, efficacy and safety by using analytical methods. According to European standards, the parameter analyzed is the measurement of the specific binding capacity of IgE immunoglobulins. The second step is to make comparisons between allergen extracts from the same allergen, but from different manufacturers [140]. Homologous allergens are established based on the identity of the amino acid sequences of the allergenic proteins [141].

## 4. Geographical Distribution and Seasonality

Through studies conducted in different regions, climates and environments, the selected taxa are proven to be significant components in the atmosphere, either by the high concentration of spores or by their constant character. The geographical distribution of the taxa analyzed confirms their ubiquity, being identified from tropical areas to polar regions. Although the presence in the atmosphere varies depending on the season, the genera *Cladosporium* and *Alternaria* remain the dominant constants [111,142,143].

*Alternaria*, *Epicoccum*, *Torula* and *Cladosporium* spores were recorded under conditions of high temperature and low humidity [144]. In Portugal, a one-year seasonal distribution analysis of the city of Porto identified high concentrations of *Alternaria* and *Cladosporium* spores during autumn and winter. The most abundant taxa in the atmosphere were *Cladosporium* (74.5%), *Aspergillaceae* (2.9%) and *Alternaria* (1.3%) [145]. In Granada, Spain, 93.82% of the identified fungal spores belonged to the genus *Cladosporium* and a strong correlation was obtained between temperature and light and the concentration of spores belonging to the dominant genera [146]. In Bratislava, of the 53 airborne fungal taxa, *Cladosporium* spores were the most frequent. The highest concentrations were recorded in summer and autumn [147]. In France, a study conducted over a 4-year period indicates, among the dominant taxa in the atmosphere, *Alternaria* and *Cladosporium* [148].

A study conducted in Singapore over a 5-year period records increased concentrations of *Cladosporium* and *Pithomyces* spores [149]. The study conducted by Kumar & Attri, 2016 [150] over a period of 14 months, in a region of the Himalayas, records the presence of spores belonging to the fungi of the genera *Cladosporium*, *Alternaria*, *Torula*, *Pithomyces*, *Epicoccum*. In Kuwait, high concentrations of *Cladosporium*, *Alternaria*, *Aspergillus*/*Penicillium* were recorded [151]. In Wuhan, China, the genera *Cladosporium*, *Aspergillus*, *Alternaria*, and *Penicillium* predominate [152].

A significant number of the United States population are sensitized to fungal aeroallergens including *Aspergillus*, *Epicoccum*, *Cladosporium*, *Penicillium*, and *Alternaria* [153]. In Havana, Cuba, between November 2010 and October 2011, the predominant taxon in the atmosphere was *Cladosporium*, with 148,717 spores identified, followed by *Aspergillus*/*Penicillium* spores [154]. In La Plata, Argentina, *Alternaria* and *Cladosporium* predominate in the atmosphere [155].

In Antarctica, the study by Gonçalves et al., 2017 [156], identified the presence of opportunistic genera *Penicillium*, *Rhodotorula* and *Cladosporium* on rocks in cold climates. In the ice cap, the genera *Cladosporium*, *Penicillium* and *Epicoccum* were identified, and species belonging to *Penicillium* and *Rhodotorula* were isolated from snow samples. [157]. In South Africa, the study by Ajikah et al., 2023 [158], shows that in Johannesburg the dominant fungal genera in the atmosphere include not only the dominant taxa *Penicillium/Aspergillus*, *Alternaria* and *Cladosporium*, but also the undercharacterized ones: *Torula* and *Epicoccum*. The study by Ndjindji et al., 2023 [159], regarding allergic sensitization in adults and children in Africa indicates allergic sensitization to the dominant taxa *Aspergillus*, *Cladosporium*, *Penicillium* and *Alternaria*. In Australia, studies indicate an increased prevalence of *Cladosporium* and *Alternaria* spores in the atmosphere [160,161].

The broad geographical distribution of atmospheric fungi, both dominant and undercharacterized, suggests that exposure to fungal spores represents a global environmental and public health issue. Although genera such as *Cladosporium*, *Alternaria*, *Aspergillus* and *Penicillium* remain dominant in most regions, studies from different climatic areas also indicate the constant presence of lesser explored fungi including *Epicoccum*, *Pithomyces*, *Torula* and *Rhodotorula*. Climatic variations may influence not only the concentration of airborne spores, but also the seasonal dynamics and regional prevalence of different fungal genera. In this context, environmental changes may contribute to modifications in exposure patterns and sensitization profiles, particularly in urban populations exposed simultaneously to air pollutants and mixed fungal aeroallergens. The increasing detection of undercharacterized taxa in geographically distant regions may also suggest that their clinical importance is currently underestimated.

## 5. Impact on Public Health

It is estimated that, globally, 30% of the population suffers from hypersensitivity, and between 3% and 10% suffer from an allergy caused by fungal spores [162,163]. Sensitization can begin as early as childhood, and the clinical course depends on the living environment, the degree of urbanization, the workplace and climatic conditions [162,164,165]. Studies show that bioaerosols may represent a risk factor for people with a weak immune system [166]. Inhalation of indoor or outdoor air exposes the body to airborne fungi, which can lead to the onset of respiratory pathology [167].

In the outdoor environment, the correlation between the increase in the concentration of spores in the atmosphere and the exacerbation of asthma symptoms is positive. In the fall, for example, the number of doctor visits due to pediatric asthma increases, and during storms, asthma attacks are more frequent [165,168]. Due to air circulation, the main source of indoor fungi is outdoor air. Fungi reach buildings through ventilation and heating systems or through contamination of materials [169]. The phrase “Sick Building Syndrome” refers to the phenomenon that occurs when a person develops unusual symptoms while working in a certain space. Symptoms include wheezing, irritation of the mucous membrane of the eyes and rhinitis, and conditions that favor the growth of fungi in indoor environments are high humidity and temperature [167,170]. Early exposure to interior dampness is often associated with the onset of asthma, and people who live in such environments are at increased risk of developing the disease [168]. Epidemiologically, indoor exposure to musty odors and dampness is directly correlated with the development of allergic rhinitis [171]. Hypersensitivity pneumonitis occurs following recurrent exposure to airborne allergens and is characterized by dyspnea, fever, and in the chronic stage can lead to pulmonary fibrosis [172].

Although indirect, the impact that fungi have on public health can also be inferred from general and medical interest, reflected in the quantity and quality of studies published in the last two and a half decades. In the following part, a bibliometric analysis of articles published between 2000 and 2025 will be presented. The databases used for the searches are Google Scholar (excluding citations and patents) and PubMed. The period of interest chosen was the interval 2000–2025. Specific search terms were used, combined in English, in the form: “fungal airborne allergens”, “[genus] fungi allergen”, where the genera analyzed were: *Alternaria*, *Cladosporium*, *Aspergillus*, *Penicillium*, *Epicoccum*, *Pithomyces*, *Torula* and *Rhodotorula*. Table 6 lists the taxa of interest and the corresponding search term. For each search term, the total number of results returned by each database and the annual distribution of publications were recorded. The data obtained were centralized using Microsoft Excel and graphically represented to highlight temporal trends and differences between Google Scholar and PubMed, as detailed in the subsections below.

### 5.1. Quantitative Trends

**Figure 1:**
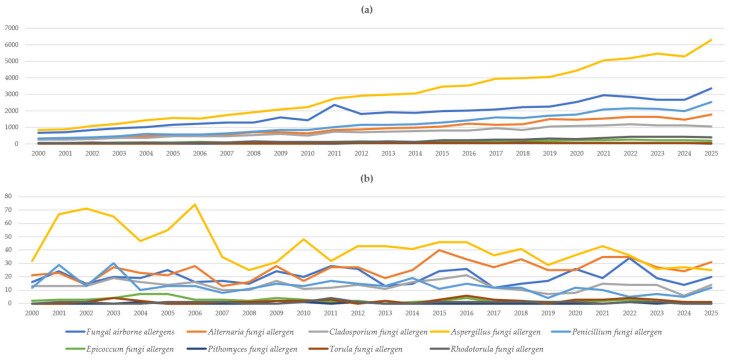
Temporal distribution of articles in Google Scholar (**a**) and PubMed (**b**).

**Figure 2:**
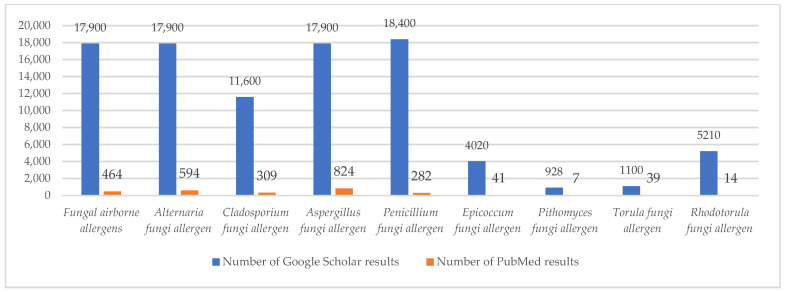
General number of articles for each search term in the 2000–2025 time period.

The search term “Fungal airborne allergens” generated 17,900 results in Google Scholar and 464 in PubMed, for the period 2000–2025 (Figure 1). While in Google Scholar the trend is upward (y = 93.886, with a strong correlation), in PubMed the number of articles fluctuates. For the dominant genera of fungi, Google Scholar shows an increasing trend, while the emerging genera mostly stagnate. Although the number of PubMed studies is lower, the same pattern of dominance can be observed. Moreover, the difference between the number of results is astonishing, going from 17,900 of articles referring to *Alternaria* in Google Scholar, to 928 articles about *Pithomyces* (Figure 2).

Academic interest in the *Alternaria* taxon is supported quantitatively and qualitatively by the increase in the number of articles in Google Scholar. The trend in PubMed is positive, but almost stagnant. With a lower number of articles for *Cladosporium* than for *Alternaria*, Google Scholar data indicates a quantitative increase in publications over time, while PubMed records a slight decrease, but without statistical relevance (R^2^ = 0.0627).

The search for the terms “*Aspergillus fungi allergen*” yielded a considerable volume of literature in both Google Scholar and PubMed; however, the upward trend in Google Scholar is not maintained in PubMed. The taxon *Penicillium* is the one with the highest number of articles recorded in the analysis for Google Scholar. In PubMed, the trend is decreasing, but without statistical relevance (R^2^ = 0.0709).

The growth of articles on *Epicoccum* was slightly fluctuating between 2000 and 2025. Quantitatively, however, the discrepancy between *Epicoccum* and the dominant genera is significant. In the case of the genus *Torula*, the number of articles is reduced, but with an upward trend in Google Scholar. In PubMed, a trend of stagnation is indicated, but with low statistical value, the value of R^2^ being 0.1181. In the case of *Rhodotorula*, there is a slight increase in the number of articles in Google Scholar, while in PubMed the trend is stagnant. The taxon with the lowest number of results is *Pithomyces*. Although there is a slight increase in Google Scholar, there are only 7 articles in PubMed.

### 5.2. Qualitative Trends

**Figure 3:**
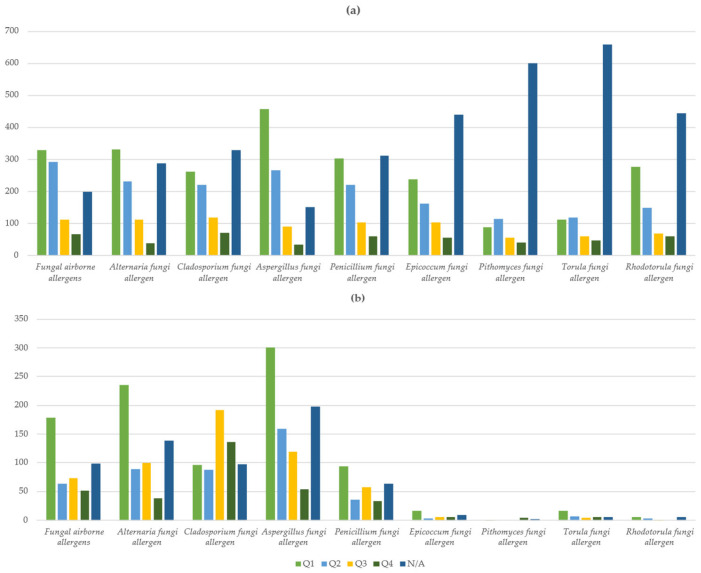
Quartile distribution of articles in Google Scholar (**a**) and PubMed (**b**).

The qualitative distribution of studies (Figure 3) sustains the undercharacterization of the emerging genera, most articles from Google Scholar being unindexed, compared to the dominant genera, where Q1 is frequent. From a qualitative point of view, the published studies for the general term “*Fungal airborne allergens*” belong predominantly to the Q1 quartile for both search engines, indicating a rigor of the published literature. For *Alternaria*, 33% of the articles in Google Scholar and 39% in PubMed belong to the Q1 quartile, indicating the dominance of specialized literature in both the public and medical fields. In the case of *Cladosporium*, the predominant percentage in Google Scholar belongs to articles indexed in quartiles, and in PubMed the dominant quartile is Q3. The data show heterogeneity in literature quality. The *Aspergillus* genera had most of the articles in the first quartile, indicating a high-quality body of literature. The distribution is heterogeneous in Google Scholar for *Penicillium*, with both Q1 and unindexed articles covering the same percentage, while PubMed shows predominance of Q1.

In the case of *Epicoccum*, 44% of studies in Google Scholar are not indexed, and 41% of studies in PubMed are Q1 indexed. For *Torula*, 66% of articles in Google Scholar are not indexed, and 44% of articles in PubMed belong to quartile Q1. This picture captures a more solid medical basis than the informational foundation of the general public and shows the need for in-depth research to confirm the allergenic potential discussed in the existing literature. The case of *Rhodothorula* shows that 44% of the articles in Google Scholar are unindexed, and in PubMed the distribution is equal for unindexed articles and Q1 (36% each). The last genera, *Pithomyces*, has most of the articles unindexed or indexed in Q4, indicating undercharacterization.

### 5.3. Thematic Trends

From a thematic point of view, the articles focus on the seasonal distribution of fungal spores and their role in allergic pathology. For *Alternaria*, the articles have as main themes the effect of the fungus on the body and the major allergen Alt a 1. Searches on *Cladosporium* led by articles that dealt with allergenicity of both dominant taxa, *Cladosporium* and *Alternaria*. The majority of the topics on *Aspergillus* are related to allergic bronchopulmonary aspergillosis. In the case of *Penicillium*, the topics addressed focus on the effects of allergens of different species of the genus on the body and on cross-reactivity. Articles about *Epicoccum* focus on cross-reactivity. The themes of the articles situate *Rhodotorula* and *Torula* as emerging genera with a role in hypersensitivity. Given the prevailing theme demonstrating the pathogenicity of the genus *Pithomyces* for both humans and animals, a future research direction is needed to strengthen, through quality studies, the current hypotheses.

## 6. Conclusions

Climate change in the 21st century has affected human health by altering the allergenic potential of fungi in the atmosphere. By integrating both widely studied and undercharacterized genera, this review creates a comprehensive picture of them. Due to their constant and quantitatively significant presence in indoor and outdoor environments, fungal spores cannot be excluded from the picture of hypersensitivity. Both traditional and modern methods allow their identification and effective diagnosis.

After analyzing the specialized literature from 2000–2025, it emerged that the airborne fungal species with the greatest impact on human health belong to the genera *Alternaria*, *Aspergillus*, *Cladosporium* and *Penicillium*. These have the most numerous results in both Google Scholar and PubMed, with an important percentage belonging to the Q1 and Q2 quartiles.

There is an obvious discrepancy between the dominant and emerging genera, which register a smaller number of articles and a qualitative mosaic in which non-indexed articles or belonging to the Q3 and Q4 quartiles predominate.

Although the role of fungi in allergic sensitization is a topic of interest for the general population, proven by the constant increase in articles in Google Scholar, quality medical studies are needed that provide a clear perspective on the taxa *Epicoccum*, *Pithomyces*, *Torula* and *Rhodotorula*. Future research should focus on comprehensive characterization of emerging fungal allergens, particularly on determining the allergenic structures of *Torula* species and the correlation of hypersensitivity and *Pithomyces* spores.

## Figures and Tables

**Table 1:** The main allergen structures of the *A. alternata* species.

Allergens	Biochemical Name	Description	Bibliographic References
Alt a 1 Major allergen	Beta-barrel protein with unique structure	Primary indicator of sensitization, with a stable dimeric structure. It is located in the cell wall of the spores. Once inhaled, it causes the production of antibodies in 80% of patients sensitized to *Alternaria*.	[46,47,48]
Alt a 3	Heat shock protein	It protects cells from oxidative and thermal stress. The presence in the serum of sensitized patients is 5%.	[43]
Alt a 4	Disulfide isomerase	As a recombinant protein, it reacts with specific IgE antibodies in the serum of 42% of sensitized patients.	[49]
Alt a 5	Ribosomal protein P2	Component of the ribosomal subunit involved in protein synthesis, it reacts with immunoglobulin E in the serum of 8–14% of sensitized patients.	[50]
Alt a 6 (previous name: Alt a 11)	Enolase	Recognized in the serum of 22% of patients sensitized to *Alternaria*. Important due to the high rate of cross-reactivity.	[51]
Alt a 7	YCP4 protein	Homologous to the yeast protein, it reacts with specific immunoglobulins in the serum of 7% of sensitized patients.	[52]
Alt a 8	Mannitol dehydrogenase	Recognized by the serum of 41% of patients sensitized to *Alternaria*, it is homologous to the NADP-dependent mannitol dehydrogenase belonging to the species *Cladosporium herbarum*.	[53]
Alt a 10	Aldehyde dehydrogenase	Recognized by *Alternaria* specific IgE in 2% of patients.	[49]
Alt a 12	Ribosomal protein P1	It is similar to Alt a 5, forms dimers together with P2, participating in protein synthesis.	[49]
Alt a 13	Glutathione-S-transferase	Recombinant protein with a major role in allergenicity, 14 out of 17 patients allergic to *A. alternata* showing positive reactions to the skin allergy test.	[54]
Alt a 14	Manganese superoxide dismutase	Enzyme involved in protection against oxidative stress. The serum of 11.5% of the 61 patients tested reacted positively to Alt a 14.	[39]
Alt a 15	Serine protease	Described as a panallergen, it reacted with a prevalence of 10.2% in the serum of sensitized patients.	[39]

**Table 2:** The main allergen structures of the *Cladosporium* genus.

Allergens	Biochemical Name	Species	Description	Bibliographic References
Cla c 9	Vacuolar serine protease	*C. cladosporioides*	Protein with a high degree of cross-reactivity with serine proteases from *Penicillium chrysogenum* and *Aspergillus fumigatus*	[59,60]
Cla c 14	Transaldolase	*C. cladosporioides*	Transaldolase with high cross-reactivity to which specific antibodies in the serum of 38% of patients with hypersensitivity to *Cladosporium* react.	[61]
Cla h 2	Glycoprotein	*C. herbarum*	Small allergen, existing in multiple isoallergenic forms.	[62]
Cla h 5/Cla h 4	Ribosomal protein P2	*C. herbarum*	The study conducted on 62 patients sensitized to *C. herbarum* showed that the serum of 22% reacted positively by binding specific IgE to Cla h 4.	[49]
Cla h 6	Enolase	*C. herbarum*	The study conducted on 62 patients sensitized to *C. herbarum* showed that the serum 20% of them reacted positively by binding specific IgE to Cla h 6.	[49]
Cla h 7	YCP4 protein	*C. herbarum*	The study conducted on 62 patients sensitized to *C. herbarum* showed that the serum of 22% reacted positively by binding specific IgE to Cla h 7.	[49]
Cla h 8	Mannitol dehydrogenase	*C. herbarum*	Major allergen to which 57% of patients with *Cladosporium* allergy react.	[63]
Cla h 9	Vacuolar serine protease	*C. herbarum*	*Cladosporium* allergy, the serum of 15.5% reacted positively by binding specific IgE antibodies to Cla h 9.	[64]
Cla h 10/Cla h 3	Aldehyde dehydrogenase	*C. herbarum*	The study conducted on 62 patients sensitized to *C. herbarum* showed that the serum of 38% of them reacted positively by binding specific IgE to Cla h 10.	[49]
Cla h 12	Ribosomal protein P1	*C. herbarum*	Allergen discovered in the 1990s using recombinant technology.	[60]

**Table 3:** The main allergens of the genus *Aspergillus*.

Allergens	Biochemical Name	Species	Description	Bibliographic References
Asp fl 13	Alkaline serine protease	*A. flavus*	63% binding capacity of specific antibodies indicating *Aspergillus* allergy.	[72]
Asp f 1	Miogilin family protein	*A. fumigatus*	85% binding capacity of specific antibodies indicating *Aspergillus* allergy.	[73]
Asp f 2	PRA-1 related protein	*A. fumigatus*	Associated with patients suffering from allergic bronchopulmonary aspergillosis.	[74]
Asp f 3	Peroxisomal protein	*A. fumigatus*	72% binding capacity of specific antibodies indicating *Aspergillus* allergy.	[75]
Asp f 4	Protein of unknown function	*A. fumigatus*	IgE binding to rAsp f 4 is very common in ABPA (92%).	[76]
Asp f 5	Metalloprotease	*A. fumigatus*	Of the 54 patients with ABPA, 93% showed IgE binding to rAsp f 5 in the ELISA test.	[77]
Asp f 8	Acidic ribosomal protein P2	*A. fumigatus*	Intercellular allergen, recognized by the body of patients suffering from ABPA.	[78]
Asp f 9	Cell wall-associated glycosidase Crf1	*A. fumigatus*	IgE binding to rAsp f 9 is common in ABPA (89%).	[79]
Asp f 11	Peptidyl-prolyl isomerase	*A. fumigatus*	The IgE response to rAsp f 11 is very common in patients sensitized to *A. fumigatus* (90%).	[80]
Asp f 12	Heat shock protein P90	*A. fumigatus*	The allergen plays an important role in triggering caspofungin resistance in *A. fumigatus*.	[81]
Asp f 13 (associated with Asp f 15)	Alkaline serine protease	*A. fumigatus*	Extracellular elastolytic protease plays a major role in the pathogenesis of invasive aspergillosis and increases airway hyperresponsiveness.	[82,83]
Asp f 16	Cell wall-associated glycosidase Crf1	*A. fumigatus*	IgE binding to rAsp f 16 is common in ABPA (70%).	[74]
Asp f 18	Vacuolar serine protease	*A. fumigatus*	IgE binding to Asp f 18 is common (79%) among asthmatic patients sensitized to *A. fumigatus*.	[84]
Asp f 22	Enolase	*A. fumigatus*	30% of asthmatic patients sensitized to *Penicillium* have IgE that recognize *P. citrinum* enolase.	[85]
Asp f 27	Cyclophilin	*A. fumigatus*	IgE binding to rAsp f 27 is common (75%) among individuals sensitized to *A fumigatus*.	[86]
Asp f 34	PhiA protein in the cell wall	*A. fumigatus*	IgE binding to rAsp f 34 is very common (93%) among ABPA patients sensitized to *A. fumigatus*.	[87]
Asp f 35	Copper Zinc Superoxide dismutase	*A. fumigatus*	Of the 52 patients with asthma, 14 were positive in the test performed.	[88]
Asp n 14	Beta-xylosidase	*A. niger*	In 11% of the 171 symptomatic bakers tested, specific anti-xylanase IgE was identified.	[89]
Asp o 13	Alkaline serine protease	*A. oryzae*	Of 70 asthmatic patients, 17 showed specific antibodies to Asp o 13. Cross-reactivity with serine protease from *P. citrinum* was demonstrated.	[90]
Asp v 13	Extracellular alkaline serine protease	*A. versicolor*	Specific IgE antibodies against Asp v 13 were identified in 8 of 40 patients allergic to *A. versicolor*.	[91]

**Table 4:** The main allergens of the genus *Penicillium*.

Allergens	Biochemical Name	Species	Description	Bibliographic References
Pen b 13	Alkaline serine protease	*P. brevicompactum*	Out of 67 asthmatic patients, serum IgE reactivity to *P. brevicompactum* was detected in 11, and 10 (91%) of them recognized the Pen b 13 protein.	[95]
Pen b 26	Ribosomal acidic protein P1	*P. brevicompactum*	Allergen of interest due to the fact that, following analysis of the amino acid sequence of Pen b 26, homology with ribosomal acidic proteins P1 belonging predominantly to aeroallergens was demonstrated.	[96]
Pen ch 13	Alkaline serine protease	*P. chrysogenum*	Of 70 sera from asthmatic patients, 17 showed IgE reactivity to *P. chrysogenum* (immunoblot); 15 of these (88%) recognized the Pen ch 13 protein. The allergen could also be directly involved in the pathogenesis of asthma.	[97,98]
Pen ch 18	Vacuolar serine protease	*P. chrysogenum*	Of the 17 patients sensitized to *P. chrysogenum*, 14 (82%) showed IgE binding to Pen ch 18.	[97]
Pen 3	Peroxisomal membrane protein	*P. citrinum*	Sera from 13 (46%) of the 28 asthmatic patients sensitized to *Penicillium* showed IgE binding to Pen c 3 on immunoblot.	[99]
Pen c 19	Heat shock protein 70 kDa	*P. citrinum*	*Penicillium* allergic patients showed IgE binding to recombinant Pen c 19 and to the 70 kDa component of the *P. citrinum* extract.	[100]
Pen c 22	Enolase	*P. citrinum*	The sera of 7 (30%) of the 23 asthmatic patients sensitized to *Penicillium* showed IgE binding to the Pen c 22 protein.	[85]
Pen 24	Elongation factor 1 beta	*P. citrinum*	Seven (8%) of the 92 sera from patients with bronchial asthma showed IgE binding to rPen c 24.	[101]
Pen c 32	Pectate lyase	*P. citrinum*	By analyzing the amino acid sequence, it was concluded that Pen c 32 shows a high degree of similarity to other fungal pectate lyases.	[102]
Pen cr 26	Ribosomal phosphoprotein P1	*P. crustosum*	14 of 61 atopic sera (23%) from individuals sensitized to *Penicillium* showed positive reactions to purified Pen cr 26.	[103]
Pen o 18	Vacuolar serine protease	*P. oxalicum*	Of the 70 sera from asthmatic patients tested, 18 (26%) showed IgE reactivity to *P. oxalicum* proteins; over 80% of these recognized Pen o 18.	[97,104]

**Table 5:** Methods of quantification of fungi allergens.

Method	Principle	Advantages	Limitations
Cultivation and microscopic analysis	Growth and optical identification of spores	Accessibility	Selectiveness and subjectivity of analysis
Passive methods	Sedimentation by gravity force	Simple to apply	No standardized air volume Influenced by shape and size of the spores
Active volumetric methods	Particles inertia; filtration, impaction and impingement	Standardized air volume	Dependable on the equipment
ELISA	Quantification of antigen-antibody reactions through color changes	Measurement and differentiation of taxa	Lack of standardized extracts
FTIR	Quantification of the natural vibrations of molecular bonds	Non-invasive	Need of special equipment
fHIA	Immunofluorescence	Significant improvement in treatment	Need of special equipment
Biosensors	Detection and quantification by coupling a biomolecule to a transductor	Real-time monitoring, increased specificity and sensibility	Emerging technique that has not been clinical validated yet

**Table 6:** Searching terminology used.

Genera	Bibliographic Reference	Searching Term
*Alternaria*	[173]	*Alternaria* fungi allergen
*Cladosporium*	[144]	*Cladosporium* fungi allergen
*Aspergillus*	[66]	*Aspergillus* fungi allergen
*Penicillium*	[24]	*Penicillium* fungi allergen
*Epicoccum*	[56]	*Epicoccum* fungi allergen
*Pithomyces*	[174]	*Pithomyces* fungi allergen
*Torula*	[175]	*Torula* fungi allergen
*Rhodotorula*	[118]	*Rhodotorula* fungi allergen

## Data Availability

The original contributions presented in this study are included in the article. Further inquiries can be directed to the corresponding author.

## Abbreviations

The following abbreviations are used in this manuscript:

ABPA	Allergic Bronchopulmonary Aspergillosis
HIA	Halogen Immunoassay
ELISA	Enzyme-linked Immunosorbent Assay
FTIR	Fourier Transform Infrared
IR	Infrared
fHIA	Fluorescent Halogen Immunoassay
AIT	Allergen Immunotherapy
